# Synergistic Effects and Mechanisms of Action of Rutin with Conventional Antibiotics Against *Escherichia coli*

**DOI:** 10.3390/ijms252413684

**Published:** 2024-12-21

**Authors:** Lankun Yi, Yubin Bai, Xu Chen, Weiwei Wang, Chao Zhang, Zixuan Shang, Zhijin Zhang, Jiajing Li, Mingze Cao, Zhen Zhu, Jiyu Zhang

**Affiliations:** 1 Key Laboratory of New Animal Drug Project of Gansu Province, Key Laboratory of Veterinary Pharmaceutical Development, Ministry of Agriculture and Rural Affairs, Lanzhou Institute of Husbandry and Pharmaceutical Sciences, Chinese Academy of Agricultural Sciences, Lanzhou 730050, China; ylankun@163.com (L.Y.); baiyb1011@163.com (Y.B.); 13231656156@163.com (X.C.); weiweiwang1990@163.com (W.W.); zchao0901@163.com (C.Z.); 13483458376@163.com (Z.S.); hns_zzj@163.com (Z.Z.); lijiajing724@163.com (J.L.); 2 School of Life Sciences and Food Engineering, Hebei University of Engineering, Hanshan District, Handan 056038, China; caomignze@126.com

**Keywords:** rutin, amikacin, *Escherichia coli*, bacterial resistance, synergistic antibacterial mechanism

## Abstract

Rutin is a widely known plant secondary metabolite that exhibits multiple physiological functions. The present study focused on screening for synergistic antibacterial combinations containing rutin, and further explored the mechanisms behind this synergy. In vitro antibacterial test results of rutin showed that the ranges of minimum inhibitory concentration (MIC) and Minimum bactericidal concentration (MBC) are 0.125–1 and 0.125–2 mg/mL, respectively. However, rutin and amikacin have a significant synergistic effect, with a fractional inhibitory concentration index (FICI) range of 0.1875–0.5. The time bactericidal curve proved that the combination of rutin and amikacin inhibited bacterial growth within 8 h. Scanning electron microscopy (SEM) revealed that a low-dose combination treatment could disrupt the cell membrane of *Escherichia coli* (*E. coli*). A comprehensive analysis using alkaline phosphatase (AKP), K^+^, and a protein leakage assay revealed that co-treatment destroyed the cell membrane of *E. coli*, resulting in the significant leakage of AKP, intracellular K^+^, and proteins. Moreover, confocal laser scanning microscopy (CLSM) and red–green cell ratio analysis indicated severe damage to the *E. coli* cell membrane following the co-treatment of rutin and amikacin. This study indicates the remarkable potential of strategically selecting antibacterial agents with maximum synergistic effect, which could significantly control antibiotic resistance.

## 1. Introduction

*E. coli* is a highly adaptable and pathogenically versatile bacterial organism. It causes various human infections, including gastrointestinal illnesses and extraintestinal infections. While *E. coli* is part of the intestinal commensal flora of humans and other mammals [1], its infections are usually treated with antibiotics. Over the last few decades, antimicrobial drugs have been widely used to treat infectious bacterial diseases in human beings, aquaculture, and horticulture. These drugs can enhance the growth of agriculture and improve the yield of livestock and poultry industries. Although antibiotics have been effective in controlling infectious diseases, their widespread use has led to the emergence of antibiotic-resistant genes. Multidrug resistant (MDR) *E. coli* has increasingly been observed worldwide, leading to concerns for humans and the veterinary medicine field due to its ability to acquire virulence factors. These factors allow it to overcome host defenses and resist antibiotics, leading to significant diseases in human hosts [2].

The emergence of MDR Gram-negative strains threatens the antimicrobial effectiveness of antibiotics. Therefore, research directions over the years have focused on the discovery of new antibiotics to address the increasingly serious challenge of drug resistance. However, the development of new antibiotics is time-consuming and expensive. Therefore, the reduced efficacy of antibiotics due to resistance has forced the development of alternative and non-traditional drugs for use instead of antibiotics [3,4]. Plants serve as a vital resource for modern medicine and are a key focus in drug discovery efforts [5]. Traditional Chinese medicine, which represents a significant portion of herbal medicine, contains numerous bioactive, plant-derived compounds that have been extensively studied worldwide. This research has revealed their potential therapeutic benefits, including antidiabetic, anti-obesogenic, antioxidant, anti-inflammatory, and antibacterial properties [6,7].

Flavonoids are naturally occurring polyphenols that exhibit a wide range of significant pharmacological functions, including anticancer [8], anti-inflammatory [9], and antiviral activities [10]. Additionally, flavonoids are well-known antibacterial agents against various pathogenic microorganisms [11], such as Staphylococcus aureus, Vibrio harveyi, Pseudomonas aeruginosa, and Enterococcus faecalis. Several flavonoids, such as fisetin, phloretin, and curcumin, demonstrate inhibitory effects on biofilms of MDR Acinetobacter baumannii isolates [12]. Rutin (yellow powder), a polyphenolic natural flavonoid also known as quercetin-3-O-rutinoside or vitamin P, is found in vegetables, citrus fruits, and plant-derived beverages. Rutin offers advantages over aglycones, exhibiting cytotoxic and mutagenic effects, therefore limiting their pharmacological use. Furthermore, rutin is considered a nontoxic chemical, making it useful in biomedical applications [13]. Studies have revealed that when rutin is used in combination with antibiotics, it enhances its effectiveness against test bacteria [14]. Although rutin has demonstrated extensive antibacterial properties and synergistic effects, the mechanism of antibacterial function remains unclear.

This study aims to screen out antibiotics that have synergistic effects with rutin and further explore the mechanism of this combination in inhibiting *E. coli*. Therefore, we evaluated the in vitro antibacterial activity of rutin and amikacin using MICs, MBCs, checkerboard tests, and time–kill assays. In addition, we determined through cell wall and cell membrane integrity testing that the combined application of rutin and amikacin will fully destroy the *E. coli* cell wall and cell membrane, causing a large amount of content to leak. This study provides a reference for developing rutin into an antibacterial synergist and reducing the use of antibiotics.

## 2. Results

### 2.1. Effect of Rutin on the Growth of E. coli

#### 2.1.1. Antibacterial Activity of Rutin

We evaluated the antimicrobial activity of rutin against *E. coli*, and the results are detailed in Table 1. Rutin exhibited antibacterial activity against *E. coli* ATCC 25922 and clinical strains of *E. coli* in vitro. The MIC values of antibiotics against *E. coli* are shown in Table S1. All clinical *E. coli* tested are MDR strains. The range of MICs of rutin against *E. coli* strains was 0.125–1 mg/mL, while the range of MBCs of rutin against *E. coli* strains was 0.125–2 mg/mL. Table 2 lists the FICI results of rutin and each antibiotic, which are used to analyze the interaction between rutin and antibiotic combinations. Most of the antimicrobial action combinations demonstrated partial synergy (0.5 < FICI < 1) or additives (FICI = 1) without evidence of antagonistic effects (FICI > 4). The synergistic effect (FICI ≤ 0.5) was observed when rutin and amikacin were combined against *E. coli*. This combination demonstrated the most pronounced synergistic antibacterial effect. The combined treatment involved using the inhibitors at a concentration of 1/4 or less than what was used alone.

#### 2.1.2. Time–Kill Studies

**Figure 1:**
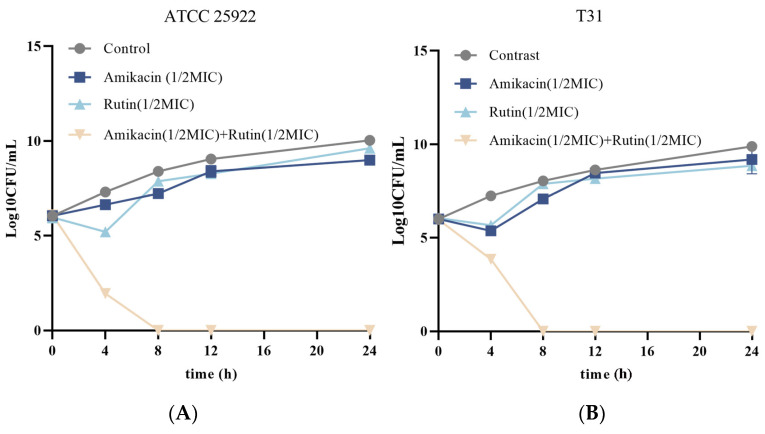
Time–kill curves of rutin and amikacin combined against *Escherichia coli*. (**A**): *Escherichia coli* ATCC 25922; (**B**): *Escherichia coli* T31.

To investigate the bactericidal performance of rutin and amikacin both individually and in combination, a time–killing kinetics assay was performed against *E. coli* ATCC 25922 and T31. As displayed in Figure 1A, the combination of rutin and amikacin displayed a faster sterilization rate compared to rutin and amikacin used alone against *E. coli* ATCC 25922. Rutin combined with amikacin achieved a bacterial reduction of more than 5 log (killing 100% bacteria) within 8 h. Similarly, the combination of rutin and amikacin completely inhibited the growth of *E. coli* T31 within 8 h (Figure 1B). These results indicated that rutin combined with amikacin exhibited rapid bactericidal kinetics against *E. coli* ATCC 25922 and *E. coli* T31, which were beneficial in preventing the development of bacterial resistance.

### 2.2. SEM Observation Results

**Figure 2:**
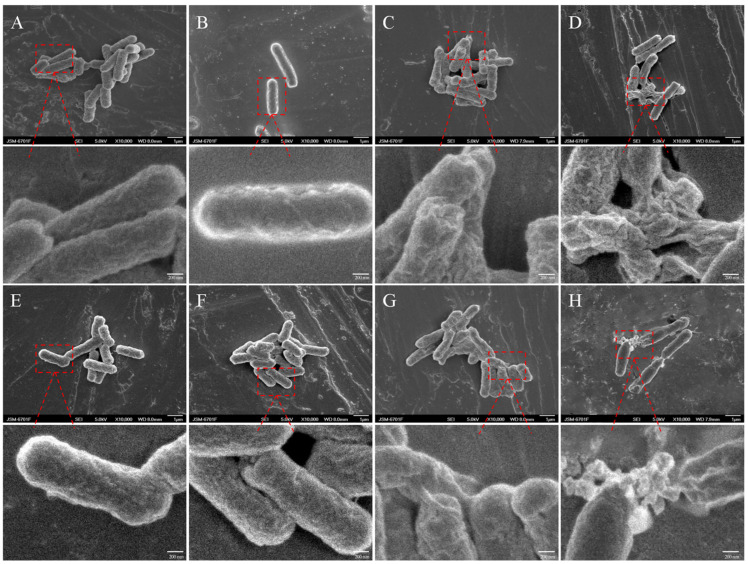
Scanning Electron Microscopy observation of *Escherichia coli* morphology (10,000×). (**A**): *Escherichia coli* ATCC 25922; (**B**): Amikacin-treated *Escherichia coli* ATCC 25922; (**C**): Rutin-treated *Escherichia coli* ATCC 25922; (**D**): Rutin and Amikacin-treated *Escherichia coli* ATCC 25922; (**E**): *Escherichia coli* T31; (**F**): Amikacin-treated *Escherichia coli* T31; (**G**): Rutin-treated *Escherichia coli* T31; (**H**): Rutin and Amikacin-treated *Escherichia coli* T31. The red parts are the representative change of the pictures.

Morphological characteristics of the bacterial cell surface of *E. coli* ATCC 25922 (Figure 2A–D) and T31 (Figure 2E–H) were examined using SEM before and after treatment with rutin and amikacin. Untreated cells exhibited an intact and smooth surface without any visible holes in the membrane or cellular debris (Figure 2A,E), indicating that the bacteria were healthy. The external morphology of *E. coli* treated with amikacin (Figure 2B,F) was similar to that of the untreated group. However, after the treatment with peptides for 6 h at a concentration of 1/2 MIC rutin + 1/2 MIC amikacin, the shapes of the *E. coli* ATCC 25922 (Figure 2D) and T31 cells (Figure 2H) were perturbed and the cells were broken. The contents of the bacteria leaked out, and the cells appeared wrinkled. The treatment of 1/2 MIC rutin resulted in shrinkage of the cell surface, while the intact structures of *E. coli* ATCC 25922 (Figure 2C) and T31 (Figure 2G) were visible in the field of view. The extent of cell disruption was less severe after treatment with 1/2 MIC rutin alone than with the combination of 1/2 MIC rutin and 1/2 MIC amikacin.

### 2.3. Effect of Rutin Combined with Amikacin on the Cell Wall of E. coli

**Figure 3:**
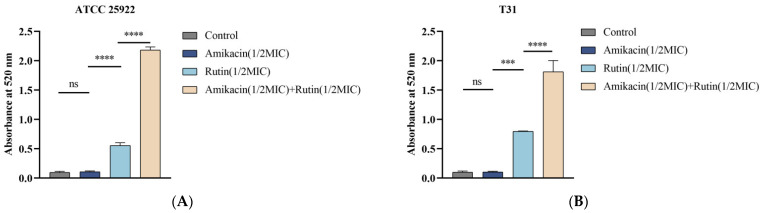
Effect of rutin and amikacin alone or combined on *Escherichia coli* Alkaline Phosphatase leakage. (**A**): *Escherichia coli* ATCC 25922; (**B**): *Escherichia coli* T31. Each value is presented as the mean ± SD (*n* = 3). ns *p*-value > 0.05, *** *p*-value < 0.001, **** *p*-value < 0.0001.

AKP is an enzyme found in the middle of the cell wall and cell membrane. Under normal conditions, AKP is not detectable outside the bacterial cell. However, when the bacterial cell wall is damaged, the cell becomes deformed or ruptured, or even dies, leading to a significant release of AKP. Consequently, AKP is an important indicator of bacterial cell wall integrity [15]. As illustrated in Figure 3A,B, AKP activity was detected extracellularly, and the amount of AKP released when rutin and amikacin were used together was significantly higher than when either was used alone (*p* < 0.0001). Additionally, there was no significant difference in AKP leakage between the group treated with amikacin and the control group (*p* > 0.05).

### 2.4. Effect of Rutin Combined with Amikacin on the Cell Membrane of E. coli

#### 2.4.1. Determination of K^+^ Leakage

**Figure 4:**
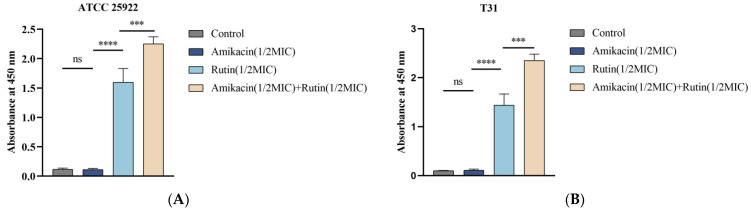
Effect of rutin and amikacin alone or combined on *Escherichia coli* K^+^ leakage. (**A**): *Escherichia coli* ATCC 25922; (**B**): *Escherichia coli* T31. Each value is presented as the mean ± SD (*n* = 3). ns *p*-value > 0.05, *** *p*-value < 0.001, **** *p*-value < 0.0001.

The leakage of K^+^ and proteins, which are essential components of bacterial cells, can occur from the cytoplasm into the culture medium. This leakage can provide insight into the integrity of the cell membrane [16]. As illustrated in Figure 4, the rutin-treated and the rutin/amikacin group significantly accelerated the membrane damage and facilitated the subsequent K^+^ leakage. Figure 4 illustrates that the rutin-combined-with-amikacin group enhanced the membrane leakage of K^+^. In the blank control group, no K^+^ was detected, and there was no significant difference between the amikacin-treated group and the blank control group (*p* > 0.05). However, K^+^ leakage from bacterial cells co-cultured with the rutin-combined-with-amikacin group was significantly higher compared to the rutin-treated groups (*p* < 0.001).

#### 2.4.2. Determination of Protein Leakage

**Figure 5:**
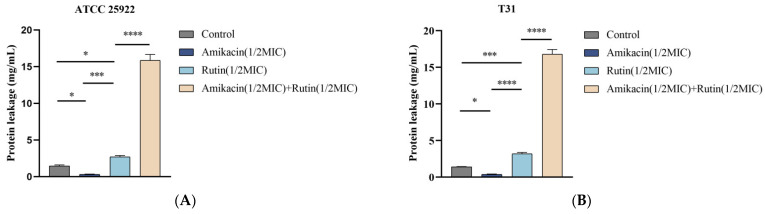
Effect of rutin and amikacin alone or combined on *Escherichia coli* protein leakage. (**A**): *Escherichia coli* ATCC 25922; (**B**): *Escherichia coli* T31. Each value is presented as the mean ± SD (*n* = 3). * *p*-value < 0.05, *** *p*-value < 0.001, **** *p*-value < 0.0001.

The protein leakage detection results are illustrated in Figure 5. The combined treatment group caused significant damage to the bacterial cell membrane, resulting in protein leakage. The amount of protein leakage was significantly higher than that of the control group (*p* < 0.0001) and also greater than that observed when rutin and amikacin were used individually (*p* < 0.0001). Moreover, rutin may have played a significant role in the synergistic treatment, leading to protein leakage.

#### 2.4.3. CLSM Result Analysis

**Figure 6:**
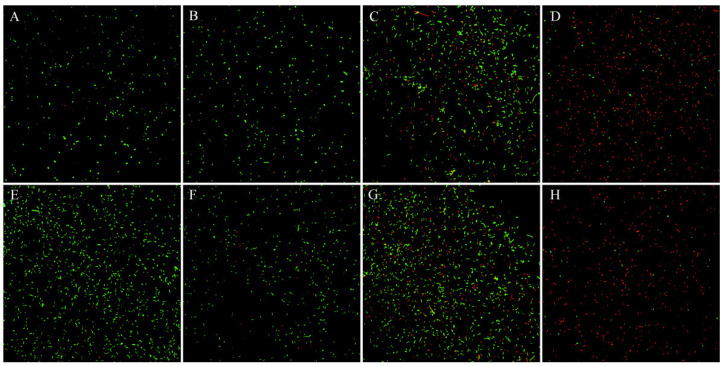
Confocal Laser Scanning Microscopy observes changes in *Escherichia coli* cell membrane integrity. (**A**): *Escherichia coli* ATCC 25922; (**B**): amikacin-treated *Escherichia coli* ATCC 25922; (**C**): rutin-treated *Escherichia coli* ATCC 25922; (**D**): rutin and amikacin-treated *Escherichia coli* ATCC 25922; (**E**): *Escherichia coli* T31; (**F**): amikacin-treated *Escherichia coli* T31; (**G**): rutin-treated *Escherichia coli* T31; and (**H**): rutin and amikacin-treated *Escherichia coli* T31. Scale Bar: 50 µm.

**Figure 7:**
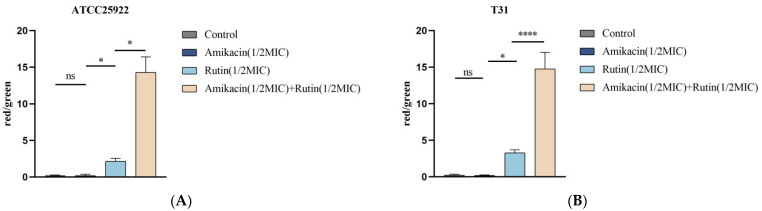
Analysis of the red–green ratio of bacteria in Confocal Laser Scanning Microscopy results. (**A**): *Escherichia coli* ATCC 25922; (**B**): *Escherichia coli* T31. Each value is presented as the mean ± SD (*n* = 3). ns *p*-value > 0.05, * *p*-value < 0.05, **** *p*-value < 0.0001.

CLSM was used to observe changes in the cell membrane integrity of *E. coli* incubated with rutin and amikacin. The live bacteria were stained with 5,6-cFDA, capable of entering intact cell membranes and emitting green fluorescence. PI was used to stain bacteria with incomplete cell membranes and emits red fluorescence. Representative pictures from the CLSM results are shown in Figure 6. As illustrated in Figure 6, most of the *E. coli* treated with amikacin emitted green fluorescence, indicating that the *E. coli* cell membrane was not damaged. In contrast, *E. coli* treated with rutin and the combination of rutin and amikacin had more red cells, indicating a disruption of the cell membrane. Moreover, the combination group resulted in a greater proportion of red blood cells than the rutin group. The analysis of the ratio of red to green cells is presented in Figure 7. The results revealed that the ratio of red to green cells in the rutin-and-amikacin-combined treatment group was significantly higher than in the group treated with the drug alone (*p* < 0.05). There was no significant difference between the amikacin treatment group and the control group (*p* > 0.05). The results revealed that the combination of rutin and amikacin effectively disrupted the cell membranes of *E. coli*.

## 3. Discussion

Rutin, a natural flavonoid also known as quercetin-3-O-rutinoside or vitamin P, is widely found in vegetables and citrus fruits. It possesses various pharmacological activities, including antibacterial and antioxidant effects [17]. The antibacterial properties of rutin have been confirmed in in vitro antibacterial activity tests [18]. In our study, rutin exhibited antibacterial activity across a concentration range of 0.125–1 mg/mL against all the tested strains. Amikacin can inhibit the growth of all tested strains in the range of 2–64 μg/mL. However, there are more and more amikacin-resistant strains. Our research indicates that rutin can reduce the MIC of amikacin by 16 times when the two are combined. As illustrated in Table 3, we computed the MICs and FICIs for five additional flavonoids against *E. coli*. The outcomes indicated that rutin exhibited an outstanding synergistic antibacterial effect, ranking among the best of the natural products tested. Incorporating plant extracts such as rutin into treatments can increase the bacteriostatic potential of amikacin by improving its antimicrobial effects. This may reduce the dose of amikacin used in treatment and thereby reduce the side effects associated with its use. The potential of rutin as an antimicrobial synergist may also inhibit the spread of bacterial resistance. Therefore, we conducted an in-depth exploration of the combined action mechanism of rutin and amikacin.

**Figure 8:**
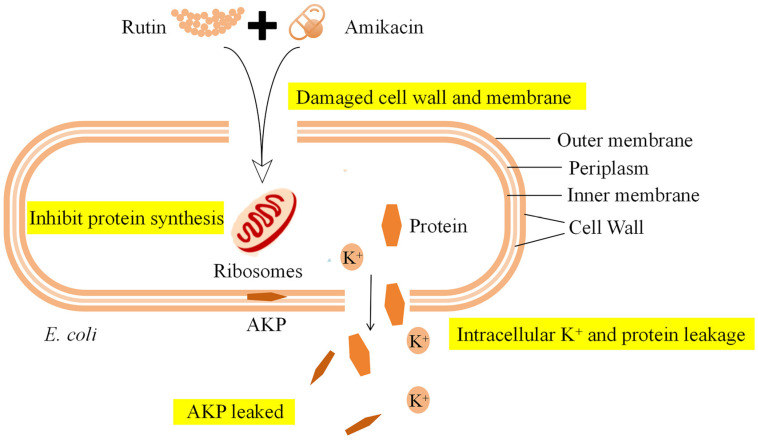
Diagram of the mechanism of action of rutin and amikacin combined against *Escherichia coli.* When rutin is combined with amikacin, it destroys the cell wall and cell membrane of *E. coli*, allowing a large amount of the drug to enter the bacteria. Amikacin inhibits ribosomes from synthesizing proteins. At the same time, AKP, intracellular K^+^, and proteins are leaked in large quantities, eventually causing the bacteria to lose their vitality.

Disruption of cell membranes is usually the primary mode of action of antibacterial drugs. Many studies have confirmed that flavonoids can inhibit bacterial activity by acting on bacterial cell membranes and cell walls [19]. Quercetin has been demonstrated to inhibit the growth of different Gram-positive and Gram-negative bacteria, as well as fungi and viruses. The mechanism of its antimicrobial action includes cell membrane damage and changes in membrane permeability [20]. This study revealed that combining rutin and amikacin to treat *E. coli* resulted in an increased leakage of bacterial AKP, K^+^, and protein. AKP, an intracellular enzyme, is located between the cell wall and the membrane. It can only be detected in the external environment when the cell wall is damaged [21]. The leakage of K^+^ and protein, which are cellular contents, indicates damage to the cell membrane integrity [22,23]. SEM and CLSM analysis revealed that after combined treatment with rutin and amikacin, the surface of *E. coli* shrank and the contents leaked, with a significant amount of PI entering the bacteria to emit red fluorescence. The results indicated that the combined application of rutin and amikacin destroyed the cell wall and cell membrane of *E. coli*, ultimately leading to the death of the bacteria. The mechanism of action of rutin and amikacin combined to inhibit *E. coli* is shown in Figure 8.

In summary, the combination of rutin and amikacin enhances the antibacterial effect of the drug alone. After rutin destroys the cell wall and cell membrane of *E. coli*, amikacin enters the interior of the bacteria in large quantities and inhibits ribosomes from synthesizing proteins, leading to bacterial death. At present, this research is still limited to the in vitro experimental part, and the in vivo antibacterial effects of rutin and amikacin still need to be explored, which is a big challenge for the clinical application of antibacterial synergists. Natural products are expected to replace antibiotics and become new drugs for the treatment of bacterial infections, or they may be used as antibacterial synergists in clinical treatment in combination with antibiotics to achieve the strategic goal of reducing and replacing antibiotics.

**Table 3:** MICs and FICIs of 6 flavonoids against *E. coli*.

	MIC (μg/mL)	FICI
Rutin	125	0.1875 (Amikacin)
Quercetin	128	0.5 (Tetracycline) [24]
Luteolin	128	0.31 (Gentamicin) [25]
(−)-epigallocatechin-3-gallate	32	0.25 (Ampicillin) [25]
Kaempferol	>512	0.258 (Colistin) [26]
Resveratrol	2048	0.254 (Colistin) [27]

MIC, Minimum Inhibitory Concentration; FICI, Fractional Inhibitory Concentration Index; *E. coli*, *Escherichia coli*.

## 4. Materials and Methods

### 4.1. Materials and Bacterial Strains

*E. coli* ATCC 25922 was procured from the American Type Culture Collection. *E. coli* strains T31, T32, T33, T34, T35, T36, T37, T38, and T39 were preserved in our laboratory. Mueller Hinton Broth (MHB), Luria-Bertani (LB), and Mueller Hinton Agar (MHA) media (Guangdong Huankai Microbial Sci. & Tech. Co., Ltd., Guangzhou, Guangdong, China) were used to cultivate all *E. coli* strains. Rutin was obtained from Shanghai Aladdin Biochemical Technology Co., Ltd. (Shanghai, China). Ceftriaxone, Amikacin, Gentamicin, Aztreonam, Meropenem, Azithromycin, Ciprofloxacin, Tetracycline, Polymyxin, and Doxycycline were acquired from Beijing Solarbio Science & Technology Co., Ltd. (Beijing, China). Rutin was dissolved in dimethyl sulfoxide. Ceftriaxone, Amikacin, Gentamicin, Aztreonam, Meropenem, Azithromycin, Ciprofloxacin, Tetracycline, Polymyxin, and Doxycycline were dissolved in aqua destillata.

### 4.2. Determination of Minimum Inhibitory Concentration (MIC)

The MIC of rutin and antibiotics was determined using the broth microdilution method and measured using 96-well titer plates [18,28]. The microorganisms were initially grown on MHA and pre-incubated for 24 h at 37 °C. Isolated colonies were then inoculated into MHB and incubated at 37 °C with shaking at 200 rpm until the exponential phase was reached. The culture was subsequently diluted to an optical density of 0.5 on the McFarland scale and diluted 100 times. Microplate wells were filled with 100 µL of MHB containing rutin concentrations ranging from 4 mg/mL to 7.8125 μg/mL and diluted bacterial suspensions. The plate was incubated for an additional 18–24 h at 37 °C.

After reading the MIC results, 100 μL of the mixture from the transparent wells was applied to MHA for culturing to determine the MBC, and the results were observed after incubation at 37 °C for 24 h. The lowest concentration that completely killed the bacteria was defined as MBC [29].

### 4.3. Antibiotic Synergism Tests

Synergistic combinations of rutin and antibiotics were determined by the checkerboard microdilution method [30]. Rutin and antibiotics were diluted in a 96-well plate. Rutin was diluted horizontally (2-fold), and each antibiotic was diluted vertically (2-fold), ensuring that each well contained a mixture of rutin and antibiotics in different concentration ratios. The MICs of rutin and antibiotics were used as the initial concentration. Then, 100 μL sample (mixture of rutin and antibiotics at different concentrations) and 100 μL bacterial liquid (bacterial liquid grown to the logarithmic phase, adjusted to McFarland turbidity 0.5 and diluted 100 times) were added to a 96-well plate, which was incubated at 37 °C for 24 h, and then the results were observed. The value of fractional inhibitory concentration index (FICI) was calculated using the given formula.

FICI = (MIC of rutin in combination)/(MIC of rutin alone) + (MIC of antibiotics in combination)/(MIC of antibiotics alone)

The combination effects were analyzed based on the following criteria: ≤0.5 denoting synergism; 0.5 < FICI < 1 indicating partial synergism; =1 denoting an additive effect; 1 < FICI < 4 denoting indifference; and ≥4 indicating antagonism [31].

### 4.4. Time–Kill Curve Assays

The time–kill curve of the combination of rutin and amikacin was used to determine its in vitro antibacterial effect [32]. Briefly, the concentration of the two *E. coli* strains was adjusted to 0.5 McFarland turbidity, and 1 mL of samples, which are MHB (control), amikacin (1 or 2 µg/mL), rutin (62.5 or 125 µg/mL), and rutin + amikacin (1 + 62.5 µg/mL or 2 + 125 µg/mL), respectively, was added. The cultures were incubated at 37 °C with shaking at 200 rpm. Subsequently, aliquots were taken out at 0, 4, 8, 12, and 24 h, serially diluted in sterile saline, and coated on MHA plates to count colony-forming units after overnight incubation at 37 °C.

### 4.5. SEM

The SEM of *E. coli* was conducted following the modified method of Yang et al. [33]. The treated organisms (1/2 MIC rutin, 1/2 MIC amikacin, and 1/2 MIC rutin + 1/2 MIC amikacin) and control were collected (5000× *g*, 4 °C, 10 min). The samples were enriched and washed once. The precipitated bacterial cells were immersed in 2.5% (*v*/*v*) glutaraldehyde overnight at 4 °C, centrifuged, and then fixed with 2.5% (*v*/*v*) glutaraldehyde for a second time for 5 h. The cells were then gradually dehydrated with graded ethanol (30%, 50%, 70%, 80%, 90%, and 100%) for 10 min. The dehydrated cells were placed on the adhesive tape and subsequently sputter-coated with gold and examined under SEM (JSM-6701F, Hitachi, Tokyo, Japan).

### 4.6. Determination of Cell Wall Integrity

The sample preparation was conducted using the modified method described by Xu et al. [34]. *E. coli* ATCC 25922 and *E. coli* T31 were cultured in an LB medium and incubated at 37 °C and 200 rpm for 12 h. After the culture was complete, it was centrifuged at 3500 rpm for 10 min and the culture medium discarded. The bacterial pellet was then washed three times with phosphate-buffer solution (PBS, pH 7.4) and the concentration was adjusted to 0.5 McFarland turbidity. Test strains were incubated with 1/2 MIC rutin, 1/2 MIC amikacin, and 1/2 MIC rutin + 1/2 MIC amikacin for 6 h at 37 °C, with untreated *E. coli* as the control. After the mixture was centrifuged at 3500 rpm for 10 min, the supernatant was collected, and AKP content was measured at 510 nm following the guidelines of the manufacturer (Nanjing Jiancheng Bioengineering Institute, Nanjing, Jiangsu, China).

### 4.7. Determination of Cell Membrane Integrity

The integrity of the cell membrane of *E. coli* was evaluated by measuring the release of cellular constituents such as proteins and K^+^ into the supernatant of cell suspensions following the modified method described [35,36]. *E. coli* ATCC 25922 and *E. coli* T31 were cultured in an LB medium and incubated at 37 °C and 200 rpm for 12 h. After the culture was complete, they were centrifuged at 3500 rpm for 10 min and the culture medium discarded. The bacterial pellet was then washed three times with phosphate-buffer solution (PBS, pH 7.4) and the concentration was adjusted to 0.5 McFarland turbidity. Test strains were incubated with 1/2 MIC rutin, 1/2 MIC amikacin, and 1/2 MIC rutin + 1/2 MIC amikacin for 6 h at 37 °C, with untreated *E. coli* as the control. They were centrifuged at 3500 rpm for 10 min to collect the supernatant for testing. Protein leakage was detected using a Bicinchoninic Acid Assay (BCA) protein detection kit (Dalian Meilun Biotechnology Co., Ltd., Dalian, Liaoning, China), while K^+^ leakage was detected using a potassium (K^+^) turbidimetric assay kit (Wuhan Elabscience Biotechnology Co., Ltd., Wuhan, Hubei, China).

### 4.8. Analysis of Intracellular Membrane Integrity with CLSM

To visually confirm the results of the cell membrane integrity test, inner membrane damage was further detected using CLSM (LSM800, Zeiss, Jena, Germany), following the methods described by Yi et al. [37]. *E. coli* cells were treated with 1/2 MIC rutin, 1/2 MIC amikacin, and 1/2 MIC rutin + 1/2 MIC amikacin. *E. coli* cells were stained with 100 μM 5,6-carboxyfluorescein diacetate (cFDA) and 15 μM propidium iodide (PI), and incubated for 15 min at 37 °C in the dark. After the incubation was complete, they were washed three times with PBS and resuspended. The resuspended sample was dropped on a glass slide and observed with CLSM. The fluorescence intensity of 5,6-cFDA and PI was detected using excitation light of 488 nm and 565 nm, respectively.

### 4.9. Statistical Analysis

The test results were analyzed with Statistical Package for the GraphPad Prism software (version 8.0). Duncan multiple range tests were used to compare the means, and *p* < 0.05 indicates a significant difference.

## 5. Conclusions

This study shows that rutin has anti-*E. coli* activity and has a synergistic antibacterial effect when combined with amikacin. Further study of its mechanism of action showed that rutin destroyed the cell wall and cell membrane of *E. coli*, allowing amikacin to directly enter the interior of the bacteria to inhibit bacterial protein synthesis and jointly exert antibacterial effects. The combined application of rutin and amikacin not only enhances the antibacterial effect of amikacin, but also reduces the dosage of amikacin, effectively alleviates the development of bacterial resistance, and is expected to be developed as an antibacterial synergist for use in the large intestine. Bacillus infection treatment has become a new strategy to control the development of drug resistance.

## Figures and Tables

**Table 1:** MICs and MBCs of rutin against ten strains of *E. coli* (mg/mL).

	ATCC 25922	T31	T32	T33	T34	T35	T36	T37	T38	T39
MIC	0.125	0.25	0.5	0.5	0.25	1	0.5	0.25	1	1
MBC	0.125	0.5	1	1	2	1	0.5	0.5	1	2

MIC, Minimum Inhibitory Concentration; MBC, Minimum Bactericidal Concentration; *E. coli*, *Escherichia coli*.

**Table 2:** FICIs of rutin combined with different antibiotics against *E. coli*.

	ATCC 25922	T31	T32	T33	T34	T35	T36	T37	T38	T39
Meropenem	0.625	1.25	1	0.75	1	1	0.625	1	1	0.75
Aztreonam	1	0.75	1	1	0.625	1	1.5	1	1	1.125
Ceftriaxone	0.75	0.625	0.5	0.75	1	1	0.5625	1	0.5	0.625
Gentamicin	1	0.625	1.25	1	1.5	1	0.75	1	1	0.75
Amikacin	0.1875	0.3125	0.5	0.5	0.375	0.375	0.5	0.375	0.5	0.25
Ciprofloxacin	0.75	1	0.75	0.5	1	1.125	1	1.5	0.5	1
Azithromycin	0.625	1	1.0625	1	0.75	1	0.75	1	0.75	1.25
Tetracycline	1	0.75	1	0.5	1	1	1	0.625	1	1.5
Doxycycline	0.75	0.625	1	1	1.5	1	0.75	1	0.75	1
Polymyxin	1	0.75	1	0.5	1	0.75	1	0.5	1	0.5

FICI, Fractional Inhibitory Concentration Index; *E. coli*, *Escherichia coli*.

## Data Availability

The original contributions presented in this study are included in the article/Supplementary Materials. Further inquiries can be directed to the corresponding author(s).

## Supplementary Materials

The supporting information can be downloaded at: https://www.mdpi.com/article/10.3390/ijms252413684/s1.
