# Supplementary material for: Synergistic Effects and Mechanisms of Action of Rutin with Conventional Antibiotics Against Escherichia coli
Source: Int J Mol Sci. 2024 Dec 21;25(24):13684. doi: 10.3390/ijms252413684 (PMC11727727; doi:10.3390/ijms252413684)
Supplement: Supplementary file 1 [file ijms-25-13684-s001.zip › Table S1.pdf]

Table S1. MICs of 10 strains of *E. coli* against 10 antibiotics (μg/mL).

	**ATCC 25922**	**T31**	**T32**	**T33**	**T34**	**T35**	**T36**	**T37**	**T38**	**T39**
Meropenem	0.015625 (S)	0.015625 (S)	0.0078 (S)	0.0078 (S)	0.0078 (S)	0.0078 (S)	0.0078 (S)	0.0078 (S)	0.0078 (S)	0.015625 (S)
Aztreonam	0.125 (S)	0.125 (S)	0.125 (S)	0.125 (S)	0.25 (S)	0.125 (S)	0.125 (S)	0.0625 (S)	0.0625 (S)	0.0625 (S)
Ceftriaxone	0.0625 (S)	0.125 (S)	0.125 (S)	0.25 (S)	0.125 (S)	0.125 (S)	0.125 (S)	0.125 (S)	0.0625 (S)	0.0625 (S)
Gentamicin	0.5 (S)	1 (S)	1 (S)	1 (S)	1 (S)	1 (S)	2 (S)	1 (S)	0.5 (S)	1 (S)
Amikacin	2 (S)	4 (S)	4 (S)	8 (S)	2 (S)	2 (S)	4 (S)	8 (S)	2 (S)	1024 (R)
Ciprofloxacin	0.0039 (S)	32 (R)	64 (R)	128 (R)	64 (R)	32 (R)	64 (R)	64 (R)	32 (R)	8 (R)
Azithromycin	0.5 (S)	32 (R)	64 (R)	128 (R)	64 (R)	32 (R)	64 (R)	64 (R)	32 (R)	8 (S)
Tetracycline	0.5 (S)	1024 (R)	512 (R)	1024 (R)	1024 (R)	1024 (R)	512 (R)	1024 (R)	1024 (R)	512 (R)
Doxycycline	0.25 (S)	64 (R)	32 (R)	64 (R)	64 (R)	128 (R)	64 (R)	64 (R)	64 (R)	32 (R)
Polymyxin	0.5 (S)	32 (R)	64 (R)	32 (R)	32 (R)	32 (R)	32 (R)	64 (R)	32 (R)	32 (R)
